# 甲基化在肺癌研究领域的相关进展

**DOI:** 10.3779/j.issn.1009-3419.2017.09.08

**Published:** 2017-09-20

**Authors:** 少伟 张, 志强 薛

**Affiliations:** 100853 北京，中国人民解放军总医院胸外科一病区 Department of Toracic Surgery, PLA General Hospital, Beijing 100853, China

**Keywords:** 肺肿瘤, DNA甲基化, 组蛋白甲基化, 早期诊断, 表观治疗药物, 精准医疗, Lung neoplasms, DNA methylation, Methylation of Histone code, Early detection of cancer, Epigenetic agents, Precision medicine

## Abstract

肺癌愈发成为威胁中国居民生命的恶性疾病，其发病率持续攀升而治疗效果不尽如人意，因此加强其早期诊断、治疗、预后等方面的研究至关重要。以“不依赖DNA序列改变而又可遗传的基因表达改变”为特点的表观遗传学研究近年来进展迅猛，尤其在DNA甲基化及组蛋白甲基化修饰研究方面取得了一系列具有临床应用价值的研究成果，全面认识其发展现状及存在问题对下一步的研究工作及实施精准医疗意义重大。本文旨在概述DNA甲基化及组蛋白甲基化修饰在肺癌领域的进展情况，并对今后相关方向的研究做出展望。

## Abstract (en)

Lung cancer is becoming an increasing threat to Chinese residents and its incidence continues to rise while the treatment effect is far from satisfactory. Hence, it is essential to improve the level of early diagnosis, treatment, prognosis in lung cancer. An epigenetic trait is a stably heritable phenotype resulting from changes in a chromosome without alterations in the DNA sequence. The epigenetic studies, such as DNA methylation and histone methylation, are progressing rapidly in oncology research. A comprehensive understanding of its development status and existing problems is of great significance for the future research and the implementation of precision medicine. Herein, we aim to outline the progress of DNA methylation and histone methylation modification in lung cancer and make a prospect for the future research.

包括肺癌在内的绝大部分疾病的发生、发展甚至预后、转归都与遗传因素息息相关，然而经典的遗传学说——DNA决定一切，或者说基因决定表型——并不能圆满地解释人类对于疾病在这些方面的困惑。随着人类基因组计划的完成，令人困惑的诸多问题并没有如事先预料的那样得到完美的解答^[1, 2]^，遑论解决临床棘手问题了。挑战随之而来，表观遗传学说不依赖DNA序列改变而又可遗传的基因表达改变^[3, 4]^，因此得到研究人员的重视并有了更大的发展。事实上，早在1942年Waddington就提出了Epigenetics一词并给出了其含义^[5, 6]^，然而由于技术及后续遗传领域专注于DNA方面的经典遗传学研究等原因，表观遗传的研究并没有很大的发展。2003年人类表观基因组计划（Human Epigenome Project, HEP）开始实施^[6, 7]^。其中DNA甲基化与组蛋白的甲基化修饰是其两项重要内容。尤其DNA甲基化作为表观遗传学研究的热门领域，随着甲基化特异性PCR（methylation-specific PCR, MS-PCR）技术的开发，其发展得到极大助力，在肺癌相关领域，涉及到基于特定基因DNA甲基化的早期诊断、分期分级诊断、化疗药物的个体化筛选、预后判断的研究日益增多；针对甲基化转移酶的药物已开始进行临床试验并取得了良好疗效。组蛋白修饰是表观遗传学的另一重要内容，其中的甲基化修饰近年来也取得了重要进展^[8, 9]^。

## 1 DNA甲基化

DNA甲基化是指甲基供体（S-腺苷甲硫氨酸，SAM）在DNA甲基转移酶（DNA methyltransferases, DNMT）的作用下，将甲基添加到DNA分子的碱基上，以胞嘧啶-鸟嘌呤二核苷酸（CpG）中的胞嘧啶5位碳原子和甲基间的共价结合最常见^[10, 11]^，CpG中胞嘧啶由此被修饰为5甲基胞嘧啶（5mC）。DNA甲基化修饰后在离体状态表现出更强的惰性，如亚硫酸氢钠可使非甲基化的胞嘧啶转变为尿嘧啶，而不能改变甲基化的CpG中的胞嘧啶；在活体状态表现为基因表达活性的降低。因此，高甲基化状态意味着基因表达的失活/抑制/沉默^[12]^，而低甲基化状态意味着基因表达的激活/活化。早期的研究发现，肿瘤细胞的DNA甲基化状态在全基因组水平上是广泛的低甲基化，导致原癌基因的活化，基因组不稳定性增加。后期研究发现，肿瘤细胞在抑癌基因、修复基因等启动子区的甲基化状态是升高的，即高甲基化，从而导致了相应抑癌基因等的表达受到抑制^[13, 14]^。且发现肿瘤细胞的高甲基化基因多发生于启动子区的CpG岛，而正常细胞启动子区的CpG岛多处于非甲基化状态。

在包括肺癌在内的绝大多数肿瘤以及诸如阿尔兹海默病、心衰等非肿瘤性疾病的患者的基因组水平均发现了DNA甲基化的异常^[15]^。由于人类基因组包含近四万个基因，大量研究发现许多疾病均可能存在特异性的甲基化谱，甚至在疾病的不同阶段甲基化谱也不尽相同；此外，据研究肿瘤细胞发生CpG岛高甲基化的频率远高于基因突变^[16]^。因此，通过对患者样本进行特定一组基因或全基因组的甲基化水平检测，理论上可以诊断疾病、辅助进行分级分期的判断甚至筛选敏感化疗药物。

### 1.1 DNA甲基化与肺癌诊断（早期诊断、分型、分级分期）

早在2005年，德国海涅大学的Schmiemann V等在发现肺癌患者中存在*APC*、*p16*（*INK4a*）、*RASSF1A*等基因的甲基化状态异常后，就提出了利用通过甲基化检测来进行肺癌的早期诊断^[17]^。

之后，借助于以MS-PCR为代表的检测技术发展，类似的研究迅猛增加，同时发现了更多的具有相对特异性的基因，如*P16*、*RASSF1A*、*APC*、*MGMT*等^[18, 19]^。一项基于痰液样本的RASSF1A、PRDM14和3OST2联合检测研究，用于区分无瘤吸烟者和Ⅰ期非小细胞肺癌（non-small cell lung cancer, NSCLC），结果显示敏感性达82.9%，特异性达76.4%^[20]^。Kikuchi等研究发现，*DAL-1*基因的甲基化随着肺癌分期的进展而出现变化^[21]^。随着研究的深入，发现基因组的低甲基化随着细胞恶性度的增加而愈加明显，有作为病情变化的判断指标的潜力^[22]^。有*meta*分析表明常见于NSCLC的*WIF-1*基因高甲基化不仅更倾向发生于鳞癌，而且其表达与较差的临床预后同样相关^[23]^。在小细胞肺癌（small cell lung cancer, SCLC）的研究上，Saito Y等通过对28例手术的SCLC患者病理组织进行检测分析，发现CpG岛甲基化表型（CpG island methylator phenotype, CIMP）是切除后SCLC的不良预后的预测因子^[24]^。这些研究表明，通过对特定基因的DNA甲基化的结果进行分析，有望为临床医生提供早期诊断、病情分期等有益的信息。不仅如此，基于*hOGG1*基因启动子甲基化在NSCLC中频繁发生的情况，有研究人员通过检测外周血单核细胞的*hOGG1*基因甲基化水平，发现甲基化的*hOGG1*基因携带者发展为NSCLC的几率是非甲基化*hOGG1*基因携带者的2.25倍，作者认为此研究可以在将来为特定人群提供肺癌发病风险预测有较大益处^[25]^。

但是，另一方面，对于同一种基因的甲基化状态在同一种疾病或者细胞系的检测结果，不同研究人员提供的数据差异很大^[26]^。例如，NSCLC中*ATM*甲基化阳性率在三个研究中的分别是47%（49/105）、0（0/37）、19%（34/180）^[26-29]^；因预测神经胶质瘤患者对烷化剂敏感性而成为明星基因的*MGMT*，针对其在NSCLC患者的甲基化状态进行的4项研究^[30-33]^中，阳性率分别为50.4%（111/220）、30.3%（37/122）、17%（12/72）、16.6%（33/199）。关于检测结果变异较大的原因，Hongdo等^[26]^认为需要强调甲基化检测技术重要性以保证检测结果的可重复性及信度，同时提及种族差异的潜在影响。但是，作者认为MS-PCR等技术的改进使得检测的灵敏度及特异度大大提升的同时，也降低了检测结果的假阳/阴性率，最终使得结果可重复性与信度得到提高。2015年，霍普金斯大学一个研究团队就开发了一种新技术——DREAMing^[34]^，该技术可以定量分析存在于NSCLC患者诸如血浆等液基标本中的极微量（0.005%的单拷贝水平）的异质性表观等位基因，这也为更为深入的科学研究及临床应用打下了良好的基础。

总之，DNA甲基化检测为解决这些棘手的临床问题提供了新的思路与方向，但针对特定基因的测定、具体基因与疾病相关性的分析及其意义的确定，可能还有相当长的路要走。检测技术的不断发展，测定标准的严格控制或许会加快DNA甲基化检测技术实际应用于临床的步伐。

### 1.2 DNA甲基化与肺癌治疗方案的选择

众所周知，传统的肺癌化疗或辅助化疗方案的制定多是基于患者肿瘤的病理分型及化疗方案实施后患者和肿瘤反应情况来确定，但是同一病理类型的患者接受同一种化疗方案获得的实际疗效差异却很大，而且在一部分患者身上可能仅仅表现出了化疗的副作用。因此，针对具体病患的个体化医疗得到越来越多患者及医疗人员的支持。随着表观遗传相关研究的深入，人员发现了某些基因与特定药物治疗反应的敏感性。例如有研究发现*MGMT*甲基化导致的基因表达失活发生在很多神经胶质瘤患者瘤内，它的发生与患者对烷化剂的敏感程度密切相关，并能提高患者的总体生存率及疾病进展时间^[18, 35]^。

这个发现引起了科研人员的极大兴趣，在肺癌的相关领域，Hsu等^[36]^发现术后NSCLC患者的CpG岛甲基化是导致的抑癌基因*p14ARF*的表达失活和p53过表达降低的原因。后续研究中发现*RASSF1A*甲基化可作为评估NSCLC患者使用吉西他滨（gemcitabine）取得较好疗效一个独立预后因子^[37]^；而在针对靶向药物吉非替尼（geftinib）耐药机制的研究中，发现可能会使p14ARF表达增加的肺腺癌患者受益^[34, 38]^。相信这些研究结果会使更多的患者因更为精准的治疗而受益。在一个基于mRNA、microRNA和DNA甲基化标记物联合应用分析Ⅰ期肺腺癌预后的队列研究中，发现*HOXA9*启动子区的高甲基化意味着更糟糕的总体生存率和更高的肿瘤复发率；三种标记物的联合应用可以更为明确地将两个队列中的高复发危险的患者识别出来^[39]^。作者认为如果这个研究能进一步得到验证，那无疑是对肺癌传统TNM分期的挑战，未来TNM分期可能需要把患者的表观遗传变异指标纳入考虑范围。

### 1.3 DNA去甲基化药物与肺癌治疗

如前所述，传统针对肺癌的化疗效果并不令人满意，即使有了更为先进的办法去预测患者对于某种具体药物是否敏感或耐药，也并不能在很大程度上提高患者的长期生存率，况且到目前为止也只能给部分具有特定表观遗传改变的某些肿瘤亚组的患者指导治疗，尤其是当检测结果提示是耐药时，可能会给患者带来更多的担忧而非希望。然而，与经典遗传学研究的DNA序列改变具有的不可逆性不同的是，包括DNA甲基化及组蛋白甲基化修饰在内的多数表观遗传学改变是可逆的^[40]^，通过逆转DNA甲基化治疗疾病的思路为疾病治疗描绘了乐观的蓝图。

DNA甲基化的形成与维持均在DNMT作用下实现的。DNMT共3个家族，即Dnmt1、Dnmt2、Dnmt3，其中Dnmt3包括Dnmt3a、Dnmt3b、Dnmt3L。人类体内有Dnmt1、Dnmt3a、Dnmt3b^[41]^。其中Dnmt1在DNA半保留复制时催化并维持新生链的DNA甲基化状态；而Dnmt3a与Dnmt3b在胚胎发育期间发挥主要作用，催化从头甲基化。三种DNMT共同维持DNA甲基化状态，并表现为正常组织低表达与肿瘤组织中的高表达^[42]^。以DNMT为作用靶点成为药物研发的新方向。有研究发现，HOXA1甲基化在SCLC耐药细胞中表达增加，并有望成为逆转肿瘤耐药的新靶点^[43]^。

然而，最先应用于临床的去甲基化药物——氮杂胞苷与地西他滨——最初的研发并不是基于DNMT的，后来发现其去甲基化作用而分别在2004年和2006年被FDA批准用于恶性血液系统的治疗并取得了较为理想的疗效^[44, 45]^。而针对诸如肺癌等实体肿瘤的研究仍处于临床试验阶段，且主要与其他药物联合进行，如小剂量的氮杂胞苷与恩替诺特联合应用治疗晚期NSCLC不仅增加患者的生存期，同时提高肿瘤对化疗药物的反应^[46]^。而单独应用去甲基化药物受限的原因在于其大剂量应用时的细胞毒性和较短的半衰期导致的难以长期维持低浓度持续发挥作用。Guadecitabine（SGI-110）作为新的去甲基化药物，是地西他滨的前提物，其水溶液更为稳定，目前正在进行恶性血液系统疾病的临床试验，Kuang等还发现其诱导肝癌细胞对奥沙利铂敏感^[47]^。2011年进入Ⅰ期临床试验的RRx-1是一种新的表观遗传药物，可同时抑制DNMT1、DNMT3a和HDACs（histone deacetylases）表达。2015年Ⅰ期临床试验结束证明了其具有较宽安全治疗窗和良好的耐受性，目前正在招募包括罹患SCLC和NSCLC在内的受试者进行Ⅱ期临床试验^[48, 49]^。其他进展主要是与其他抗肿瘤方式联合进行治疗研究^[50]^。

总之，在表观遗传其他方面迅猛发展的大背景下，对于针对肺癌等实体肿瘤的表观药物研发进展情况并不理想。对现有药物的进一步修饰以提高药物的靶向性而降低药物不良反应是下一步需要做的工作^[45]^。作者认为药物研发的思路也许需要做一个转换，针对肿瘤全基因组水平低甲基化状态与局部基因启动子区的高甲基化状态，我们不能只针对高甲基化状态去开发去甲基化药物，针对低甲基化状态去开发促甲基化药物也是一个需要研究的方向，甚至对甲基化进行双向调节维持平衡药物进行研发也是一个方向。而且去甲基化机制的研究很可能会促使新的药物靶点发现与开发。原癌基因启动子区被诱导为高甲基化状态后基因表达失活同样可以抑制肿瘤生长，当然进一步的研究仍需要大量困难需要克服。目前，表观遗传研究领域前沿的某些专家也在呼吁关注并研究以前忽视的非编码序列^[51]^。或许，我们在甲基化药物研发上也该转换思路了。

## 2 组蛋白的甲基化修饰在肺癌领域的应用

组蛋白修饰是表观遗传学研究的另一重要内容，不同的组蛋白因为其末端氨基酸不同而出现不同类型的修饰，包括乙酰化、甲基化、磷酸化、泛素化等^[8, 52]^。其中组蛋白的乙酰化和磷酸化修饰是可逆的，而且对基因转录的作用相互拮抗。组蛋白甲基化是由组蛋白甲基化转移酶（histonemethyl transferase, HMT）完成的。不同于DNA甲基化所导致的基因表达沉默的是，组蛋白甲基化对基因表达可产生激活/抑制的作用，如*H3-K9*、*H3-K27*、*H4-K20*甲基化会抑制基因表达，而*H3-K4*、*H3-K36*、*H3-K79*甲基化则具有激活效应^[53, 54]^；而且不同类型（单/双/三甲基化）的甲基化作用也不一样。此外，组蛋白甲基化和DNA甲基化可能共同参与基因表达过程^[55, 56]^。

### 2.1 组蛋白甲基化与肺癌诊断

目前关于组蛋白甲基化的研究主要集中在基础机制领域，与临床应用相关的研究涉及相对较少，而且多是与其它相关研究共同进行。比如，基于H4KA16乙酰化缺失和H4KM20三甲基化是肿瘤普遍特点的特性，Li等研究了H4KA16、H4KM20及Ki67在肺神经内分泌肿瘤特点，发现H4KA16与H4KM20的进行性损失与肿瘤级别有关，并反映了肿瘤细胞的分化和增殖活性的程度。认为这些组蛋白修饰可以作为肿瘤生物标志物帮助临床诊断和预后评估^[57]^。H3-K9的组蛋白甲基化转移酶KMT1E/SETDB1协同TGFβ调节复合物SMAD2/3在肺癌转移上发挥抑制作用^[58]^。Ávila-Moreno等研究发现MEOX2和TWIST1的过表达与H3K27me3和H3K4me3水平相关，在前者低表达而在后者高表达，进一步研究证实可依此帮助判断NSCLC患者的化疗药物耐药性和预后^[59]^。在诊断、预后评估等方面的研究应用与DNA甲基化所做工作类似，只是研究工作没有DNA甲基化广泛。

### 2.2 与肺癌相关的调节组蛋白甲基化药物

与DNA甲基化修饰及组蛋白乙酰化/磷酸化修饰不同的是，组蛋白甲基化一度被认为是不可逆的，这就降低了其在药物开发领域的应用价值，直到2004年LSD-1（lysine specific demethylase 1）的发现证实存在组蛋白去甲基化酶（histone demethylase, HDM）后^[60]^。有研究提示抑制LSD1在SCLC临床前研究模型中显示出细胞增殖减少、肿瘤干细胞维持降低，同时促进细胞分化并抑制肿瘤生长。因此LSD1有望成为SCLC的治疗靶点以改善其不良预后^[61]^。

目前针对HDM及HMT的药物研发工作已大量开展，现主要有三类药物：第一类是EZH2（enhancer of zeste homolog 2）抑制剂，EZH2是多梳家族蛋白（polycomb-group proteins）中PRC2的催化核心的亚基；PRC2具有HMT活性，对X染色体的失活具有作用^[62]^。EZH2在包括肺癌在内的多种实体肿瘤表达过度，从而诱导肿瘤细胞的迁移、聚集，相反在正常组织极少表达，因此还有作为肿瘤标志物的潜能^[63]^。Fillmore等研究发现，通过抑制EZH2可以使具有BRG1和*EGFR*突变的NSCLC对依托泊苷敏化^[64]^。最近，Eric E. Gardner等利用EZH2抑制剂——EPZ011989，在小鼠PDX模型（patient-derived xenograft models）上成功地预防了SCLC获得性耐药的发生，并在耐药和敏感模型上均提高了疗效^[65]^。第二类是DOT1L抑制剂，DOT1L同样是一种HMT，催化H3K79的甲基化，同样有针对血液系统肿瘤的药物进入临床试验^[66]^。第三类是HDM抑制剂，如上一段提及的抑制LSD-1，现已研发出作用于LSD-1的抑制剂GSK2879552，已作为一种新的有效的高选择性可口服的药物进入SCLC的临床试验，并在体内和体外均显示出抗肿瘤活性，且DNA低甲基化特征可作为其生物活性的预测因子^[67]^。未来可能同样会根据表观遗传标志物检测结果进行药物选择并联合传统化疗药物进行抗肿瘤治疗。

## 3 展望

表观遗传学研究近十余年来蓬勃发展，尤其在肿瘤研究领域进展迅速。但仍有一些问题需要去解决：从基础医学角度来讲，肿瘤与甲基化因果关系的阐明需要有效的动物模型，动物模型建立后对肿瘤的发生机制认识与抗肿瘤药物的研发大有裨益。从流行病学角度来讲，饮食与基因组甲基化水平的关系需要得到进一步研究，低/高甲基化饮食对体内基因组甲基化水平的是否有直接影响，已经有研究团队对比了孕期高甲基化摄入孕妇与正常饮食孕妇生产后子代体内甲基化水平并得到阳性结果^[68]^，这对指导具有表观遗传变异患者的饮食大有裨益，当然这仍需要更为深入的研究。从肺癌的高危因素吸烟角度，已经有团队做了吸烟与特定基因甲基化水平的队列研究^[69]^，这对揭示DNA甲基化、吸烟、肺癌三者之间关系的研究以及肺癌的早诊与治疗均能提供很好的线索。从临床流行病学角度来讲，患有与低甲基化摄入有关的疾病（比如巨幼细胞性贫血）的患者在肿瘤的发生、发展、病理类型或表观遗传改变是否与其他人群有差异，同样需要大量工作要做。随着认识的深入，结合精准医学的概念，从甲基化角度出发，人类有希望取得更大的突破，使得包括肺癌在内的广大病患的病痛得以缓解甚至根除。
